# Chromatin remodeling in oligodendrogenesis

**DOI:** 10.18699/VJ21.064

**Published:** 2021-09

**Authors:** E.V. Antontseva, N.P. Bondar

**Affiliations:** Institute of Cytology and Genetics of the Siberian Branch of the Russian Academy of Sciences, Novosibirsk, Russia; Institute of Cytology and Genetics of the Siberian Branch of the Russian Academy of Sciences, Novosibirsk, Russia

**Keywords:** oligodendrocyte, myelination, epigenetic regulation, gene expression, олигодендроцит, миелинизация, эпигенитическая регуляция, экспрессия генов.

## Abstract

Oligodendrocytes are one type of glial cells responsible for myelination and providing trophic support for axons in the central nervous system of vertebrates. Thanks to myelin, the speed of electrical-signal conduction increases several hundred-fold because myelin serves as a kind of electrical insulator of nerve f ibers and allows for quick saltatory conduction of action potentials through Ranvier nodes, which are devoid of myelin. Given that different parts of the central nervous system are myelinated at different stages of development and most regions contain both myelinated and unmyelinated axons, it is obvious that very precise mechanisms must exist to control the myelination of individual axons. As they go through the stages of specif ication and differentiation – from multipotent neuronal cells in the ventricular zone of the neural tube to mature myelinating oligodendrocytes as well as during migration along blood vessels to their destination – cells undergo dramatic changes in the pattern of gene expression. These changes require precisely spatially and temporally coordinated interactions of various transcription factors and epigenetic events that determine the regulatory landscape of chromatin. Chromatin remodeling substantially affects transcriptional activity of genes. The main component of chromatin is the nucleosome, which, in addition to the structural function, performs a regulatory one and serves as a general repressor of genes. Changes in the type, position, and local density of nucleosomes require the action of specialized ATPdependent chromatin-remodeling complexes, which use the energy of ATP hydrolysis for their activity. Mutations in the genes encoding proteins of the remodeling complexes are often accompanied by serious disorders at early stages of embryogenesis and are frequently identif ied in various cancers. According to the domain arrangement of the ATP-hydrolyzing subunit, most of the identif ied ATP-dependent chromatin-remodeling complexes are classif ied into four subfamilies: SWI/SNF, CHD, INO80/SWR, and ISWI. In this review, we discuss the roles of these subunits of the different subfamilies at different stages of oligodendrogenesis

## Abstract (ru)

Олигодендроциты – это один из типов глиальных клеток, ответственных за миелинизацию и обеспечивающих трофическую поддержку аксонов в центральной нервной системе позвоночных. Благо- даря миелину скорость проведения электрического сигнала увеличивается в сотни раз, так как он служит своего рода электроизолятором нервных волокон и позволяет осуществлять скачкообразную передачу по- тенциала действия через лишенные миелина перехваты Ранвье. Поскольку разные части ЦНС миелинизиру- ются на различных стадиях развития и большинство регионов содержит как миелинизированные, так и не- миелинизированные аксоны, очевидно, что должны существовать очень точные механизмы для контроля миелинизации отдельных аксонов. При прохождении через стадии спецификации и дифференцировки – от мультипотентных нейрональных клеткок вентрикулярной зоны нервной трубки до зрелых миелинизирую- щих олигодендроцитов, а также во время миграции вдоль кровеносных сосудов к пункту назначения, клет- ки претерпевают кардинальные изменения в паттерне экспрессии генов. Эти изменения требуют тщательно скоординированного в пространстве и времени взаимодействия различных транскрипционных факто- ров (ТФ) и эпигенетических событий, определяющих регуляторный ландшафт хроматина. Ремоделирование хроматина существенно влияет на транскрипционную активность генов. Основной компонент хромати- на – это нуклеосома, которая, помимо структурной, выполняет регуляторную функцию и служит общим ре- прессором генов. Для изменения типа, положения и локальной плотности нуклеосом необходимо действие специализированных АТФ-зависимых комплексов ремоделирования хроматина, которые используют для своей работы энергию гидролиза АТФ. Мутации в генах, кодирующих белки комплексов ремоделирования, часто сопровождаются серьезными нарушениями на ранних стадиях эмбриогенеза и с высокой частотой идентифицируются при различных раковых заболеваниях. Большинство идентифицированных АТФ-зависимых комплексов ремоделирования хроматина классифицируется на четыре подсемейства: SWI/ SNF, CHD, INO80/SWR и ISWI, согласно доменной организации их АТФ-гидролизующей субъединицы. В настоящем об- зоре мы подробно остановимся на роли этих субъединиц разных подсемейств на различных этапах олиго- дендрогенеза.

## Introduction

Until recently, in the research on the workings of the brain, the central role in the functioning of the central nervous system has been assigned to neurons, and various pathological conditions have been regarded as a result of impaired functioning of neurons. Glial cells have been assigned the function of a filler for the space among neurons, as reflected in the name: glia is translated as “glue”. Nonetheless, at present, there is no doubt about their necessity for the maintenance of axonal functions, for synaptic plasticity, and for the formation of neural networks.

Oligodendrocytes (OLs) are a type of glial cells responsible for myelination of axons in the central nervous system of vertebrates. As a result of an extremely specialized process of intercellular interaction, each OL forms several processes, each of which repeatedly wraps a part of an axon like insulating tape. One OL can simultaneously myelinate up to 50 axonal segments (Nave, Werner, 2014). The cytoplasm is almost absent in these processes, and, accordingly, the myelin sheath is actually many layers of the cell membrane with dielectric properties; this arrangement prevents the scattering of an electrical signal traveling along the axon. In addition, between the layered wrappings (sheaths) of myelin, there are ~1 micron-wide gaps, called Ranvier nodes, which enable saltatory transmission of a nerve impulse.

Thus, due to the myelin sheath, the speed of the electrical signal along the axon increases manyfold. It has been shown that the most active axons in the brain receive heavier myelin insulation, which allows them to work even more efficiently. It should be noted that myelination of the central nervous system proceeds over a long period and is the final stage in the development of the nervous system. According to magnetic resonance imaging data from humans, the bulk of myelination of most brain structures occurs in adolescence and reaches 90 % by the age of 20–25, whereas the latematuring prefrontal cortex is the last to be myelinated (Lebel et al., 2008). In mice, most of myelination takes place in the first 4–7 weeks after birth. It is worth mentioning that myelin formation continues throughout the lifespan in the form of either remyelination of damaged nerve fibers or myelination of previously unmyelinated ones (Zhu et al., 2011; Bartzokis et al., 2012; Young et al., 2013).

Given that different parts of the central nervous system are myelinated at different stages of development and most of central nervous system regions contain both myelinated and unmyelinated axons, it is clear that very precise mechanisms must exist to control the myelination of individual axons. In addition, it is becoming increasingly obvious that there is some level of plasticity (remodeling) based on life experience in the myelination process (Mitew et al., 2014).

Specif ication, migration, and differentiation of OLs

Oligodendrocytes come into being as a result of gradual differentiation of oligodendrocyte precursor cells (OPCs) that arise during specification of multipotent neuronal progenitor cells located in the ventral zone of the neural tube. In the specification of OPCs, one of the key factors that determines this localization is the sonic hedgehog (SHH) protein secreted by the cells of the notochord and floor plate. The dorsal-ventral SHH concentration gradient promotes the formation of OPCs mainly from the ventral neuroepithelium by inducing the expression of a number of transcription factors (NKX2.2, PAX6, SOX10, OLIG1, and OLIG2). Oligodendrocyte precursor cells are characterized by the expression of proteoglycan NG2 and platelet growth factor receptor alpha (PDGFRα). In the absence of SHH in the anterior or spinal cord, OPCs of ventral origin do not form (Orentas, Miller, 1996; Pringle et al., 1996; Tekki-Kessaris et al., 2001). After specification, OPCs proliferate and migrate along blood vessels, ensuring an even distribution of white matter in the central nervous system (Dejana, Betsholtz, 2016; Tsai et al., 2016). After the migration of OPCs, some of them remain in the precursor state, while others – through the stage of premyelinating OLs – differentiate within at least 2–3 days into mature myelinating cells, which interact with axons and give rise to myelin internodes (Zhu et al., 2011; Mitew et al., 2014).

To avoid a shortage of OLs during axon myelination, an excess of these cells is generated, and unnecessary cells are subsequently eliminated by apoptosis. One of the mechanisms that determine the final OL number is competition for a limited amount of specific growth and survival factors, such as platelet growth factor (PDGF)-A, fibroblast growth factor (FGF)-2, insulinlike growth factor (IGF)-1, neurotrophin (NT)-3, and ciliary neurotrophic factor (CNTF) (Barres, Raff, 1994; Miller, 2002).

As expected, the differentiation into OLs should be consistent with a neuronal development program, and there are neuronal signals that control the conversion of OPCs into myelin-forming OLs. For instance, in the peripheral nervous system, on the axonal membrane, there is a protein called neuregulin 1, which controls myelination by Schwann cells; however, a similar regulatory protein triggering the myelination by OLs in the central nervous system has not yet been identified. Moreover, there is evidence that there are inhibitory neuronal signals that keep OPCs in a “suppressed” state (Emery, 2010). These inhibitory signals (for example, Jagged, PSA-NCAM, and LINGO-1) coming from axons in turn activate various regulators of transcription, such as SOX5/6, HES5, and ID2/4, which actively prevent OPCs from entering the stage of terminal differentiation (Piaton et al., 2010; Taveggia et al., 2010).

More and more data indicate that different areas of the central nervous system correspond to different OPC populations that are controlled by some local signaling mechanisms. Thus, in different areas of the brain and spinal cord, OPCs are under the influence of different signaling molecules. In particular, dissimilarities in the temporal expression of these factors and signals in a developing central nervous system ensure that myelination begins earlier in the spinal cord and later in cortical regions.

When going through the stages of specification, migration, proliferation, and differentiation, OLs undergo dramatic changes in the pattern of gene expression, which require a precisely (temporally and spatially) coordinated interaction of various transcription factors and epigenetic events that determine the regulatory landscape of chromatin (Copray et al., 2009). Chromatin remodeling considerably affects transcriptional activity of genes and occurs mainly due to nucleosome repositioning (under the action of ATP-dependent chromatin-remodeling complexes), chemical modifications of histones, DNA methylation, and interactions with noncoding RNAs ( Gregath, Lu, 2018; Koreman et al., 2018).

In the present review, we will examine in detail the functions of ATP-dependent chromatin-remodeling complexes at various stages of oligodendrogenesis.

ATP-dependent chromatin-remodeling complexes

The regulation of gene expression is possible for the most part due to changes in the structure of chromatin: its remodeling. The main component of chromatin is the nucleosome, which consists of eight protein subunits (histones), wrapped by a 146 bp DNA region for each nucleosome. In addition to the structural function (there is growing evidence that it is less important), the nucleosome performs a regulatory function and serves as a general repressor of genes. It interferes with virtually all DNA-related processes, including transcription, replication, and DNA repair (Kornberg, Lorch, 2020). Changes in the type, position, and local density of nucleosomes require the action of specialized ATP-dependent chromatin-remodeling complexes (so-called remodelers), which use the energy of ATP hydrolysis for their functioning.

All ATP-dependent chromatin-remodeling complexes contain an ATP-hydrolyzing subunit belonging to the SNF2 DNA helicase/translocase family and one or more subunits that are bound to it (Hota, Bruneau, 2016). It should be noted that these ATPases lack helicase activity for strand unwinding and therefore act only as DNA translocases (Dürr et al., 2005) that move DNA along the surface of the nucleosome and disrupt DNA–histone interactions. ATPases of all chromatin remodelers contain a highly conserved core ATPase domain, which is sufficient for the catalytic activity of the complex; however, ATPase activity regulation is carried out by the domains flanking the ATPase domain and/or by proteins physically associated with it (Clapier et al., 2017). It must be pointed out that tissue specificity of the subunits of a chromatin-remodeling complex gives it unique properties, which determine its landing on tissue-specific regulatory loci of the genome (Hota, Bruneau, 2016).

According to sequence homology of the central ATP-hydrolyzing subunit, most of the ATP-dependent chromatinremodeling

complexes identified so far are classified into four subfamilies: SWI/SNF (switch/sucrose nonfermentable), CHD (chromodomain helicase DNA-binding), INO80/SWR (Inositol-requiring 80/SWi2/snf2-related 1), and ISWI subfamily (imitation switch). Aside from differences in the number of subunits in the complex, these subfamilies are distinguished by exclusive domains adjacent to the core ATPase region. For instance, SWI/SNF is characterized by the presence of a bromodomain, CHD – by tandem chromodomains, ISWI remodelers contain HSS helicase domains, and INO80/SWR members have an HSA domain (SANT helicase) (Bartholomew, 2014; Clapier et al., 2017).

The structural differences between the subfamilies determine their functionality. For example, members of the SWI/SNF subfamily are mainly responsible for the removal and repositioning of nucleosomes, thereby providing access to DNA for transcription factors (in order to regulate the level of gene expression) and for DNA repair and recombination factors. The assembly and positioning of nucleosomes is mainly conducted by remodelers of subfamilies ISWI and CHD. The editing of nucleosomes, namely the replacement of canonical histones with specialized types and vice versa, is carried out mainly by members of the INO80 subfamily. Changing the composition of nucleosomes allows for the creation of specialized regions of chromatin in a replication-independent manner (Venkatesh, Workman, 2015; Clapier et al., 2017). For example, histone H2A.Z is a part of the nucleosomes located in promoter regions of most genes; the proximity of such a nucleosome to a transcription start site directly correlates with the expression level of the gene in question (Bargaje et al., 2012), H2A.X is required for the repair of double-strand breaks (Elsesser et al., 2019).

Mutations in the genes encoding subunits of ATP-dependent chromatin-remodeling complexes are often accompanied by severe abnormalities in the early stages of embryogenesis and are frequently identified in various cancers (Ho, Crabtree, 2010; Wilson, Roberts, 2011).

Remodelers’ actions during oligodendrogenesis

Subfamily SWI/SNF

Mammalian members of SWI/SNF are large complexes of ∼1.5 MDa and consist of at least 15 different subunits (Hota, Bruneau, 2016). They are characterized by the presence of enzyme Brm (encoded by the Brahma gene, also known as Smarca2) or Brg1 (Brahma-related gene 1, aka Smarca4) as core translocases. These remodelers are mainly involved in the regulation of the cell cycle and differentiation of several cell types (Matsumoto et al., 2016).

A comparative analysis of genome-wide chromatin immunoprecipitation- high-throughput sequencing (ChIP-seq) data on the localization of RNA polymerase RNAPII on the genomic DNA of OLs at different stages of differentiation (rat primary OPCs in culture, immature OLs, and mature OLs) has revealed significant enrichment in genes coding for proteins of the SWI/SNF remodeling complex during the transition from OPCs to immature OLs (Yu et al., 2013). In particular, in response to differentiation signals, a significant increase in the RNAPII amount was registered on exons of the Brg1 gene (encoding the core ATPase of the SWI/SNF complex) but was not observed on the gene of its only homolog, Brm (Yu et al., 2013).

During early embryonic development (embryonic day 12; E12), Brg1 transcripts are virtually undetectable in proliferating neuronal stem cells (NSCs) of the ventricular zone (VZ) in the mouse forebrain, in contrast to the already differentiated postmitotic cells of the mantle zone (Randazzo et al., 1994). This pattern of Brg1 expression changes after E13, in such a way that most VZ cells become Brg1-immunoreactive (Matsumoto et al., 2006), in good agreement with the data on the beginning of the first wave of OPC specification in mice on E12.5 during this period (Cai et al., 2005). A Cre-mediated knockout involving a conditional deletion of Brg1 under the control of nestin (an NSC marker) in mice leads to a decrease (in the ventricular zone, E13.5) in the number of proliferating NSCs capable of subsequently undergoing gliogenesis. It is assumed that this phenomenon can be mediated by stem cell differentiation into postmitotic cells (neurons) and/or by apoptosis. Those authors believe that Brg1 keeps NSCs in an undifferentiated state until they respond to some gliogenic signals during development in mammals (Matsumoto et al., 2006).

An increase in the transcription of Brg1 has been noted exclusively during the transition from precursors to immature OLs and is not observed during differentiation of other cell types, suggesting that this is a unique event initiating oligodendrocytic differentiation. Western blotting and immunohistochemical results also indicate that Brg1 expression is mainly limited to differentiating oligodendrocytic cells (Yu et al., 2013). By contrast, in another study, according to immunohistochemical findings, Brg1 expression was detectable at all stages of OL development, and there was no obvious difference in staining intensity between OPCs and maturing OLs (Bischof et al., 201

In mice with a conditional knockout of Brg1 (with Cre under the control of Olig1, the expression of which starts with the formation of OPCs), a myelination-deficient phenotype manifests itself, although the number of OPCs and their proliferation rate are comparable to those in control mice. It turns out that these mice feature increased expression levels of differentiation inhibitors (ID2/4, Nfia/b, Sox5, and β-catenin) and downregulation of genes associated with both the synthesis of lipids and myelin sheath proteins and with differentiation regulation (Gm98/MRF and Sox10) (Yu et al., 2013). In the aforementioned work of M. Bischof and coworkers (2015), it was also found that early deletion of Brg1 (with Cre under the control of Brn4 expressed in NSCs) throughout the ventricular zone of the spinal cord also prevents Sox10 expression in OLPs until the end of embryogenesis.

ChIP-seq with antibodies to Brg1 revealed an order of magnitude more peaks in immature OLs than in OPCs, while almost all the peaks in OPCs overlapped with the peaks of immature OLs but had significantly lower intensity. Gene analysis showed that Brg1 target genes are mainly associated with myelination, oligodendrocytic differentiation, and cell cycle arrest (e. g., Cnp, Cldn11, Klf9, and Zfp191). Those authors hypothesized that Brg1 mainly targets intergenic enhancer regions because ~80 % of peaks of Brg1 binding in immature OLs were located at a considerable distance from a transcription start site (Yu et al., 2013).

Transcription factor Olig2 is thought to play an important role in the regulation of Brg1 functions in oligodendrogenesis. It is reported that Olig2 not only regulates Brg1 expression but also recruits the SWI/SNF complex with the Brg1 ATPase to OL-specific enhancers during the critical transition from OPCs to immature OLs; as a consequence, directed chromatin remodeling ensues, which is necessary for activation of the oligodendrocytic differentiation program (Yu et al., 2013).

Moreover, it has been revealed that at early stages of development (E14.5), Brg1 within the SWI/SNF complex interacts with the proximal promoter of the Olig2 gene in cortical NSCs and suppresses its expression, whereas in cells of the ventricular zone, where oligodendrocytic differentiation is seen first, no suppressive effect of Brg1 on Olig2 was found (Matsumoto et al., 2016).

Therefore, all these data indicate that Brg1, which is a part of the SWI/SNF complex, is required for both OPC specification and oligodendrocytic differentiation

Subfamily CHD

ATPases of this subfamily of remodelers are represented by nine proteins, CHD1–9, which differ in their domain organization. The complexes formed by them can combine 1 to 10 subunits. Some of them shift or push nucleosomes to facilitate transcription, whereas others play a repressive part (Clapier, Cairns, 2009), and thus are important regulators of cell differentiation (Martin, 2010). It has been demonstrated that chromodomain ATPases Chd7 and Chd8 are important for the initiation of the processes of nerve-fiber myelination as well as remyelination in the pathogenesis of some disorders (He et al., 2016; Doi et al., 2017; Marie et al., 2018).

Chd7 expression is present in most of oligodendrocytic cells in a number of structures within a developing brain (postnatal day 14, PND14) and in the spinal cord. A substantial proportion of these cells are differentiated OLs with Chd7 overexpression, whereas in OPCs, Chd7 expression is reported to be lower (He et al., 2016). A conditional knockout of the Chd7 gene using the Olig1–Cre construct reduces the number of OLs expressing MBP both at embryonic and early postnatal stages of development and decreases the expression of basic myelin proteins Mbp and Plp1 at the mRNA level. As a result, in the mutant animals, there is a decrease of white-matter volume in the brain and impaired myelination of nerve fibers (a lower g-ratio) in comparison with control animals (PND14). In the cerebral cortex, the Chd7 deletion also leads to a decrease in the number of mature OLs at the early postnatal stages of development but does not have a significant impact on the number of OPCs and their proliferation. Nevertheless, with age, mutant mice showed a gradually rising number of mature OLs, and by PND60, the degree of myelination in the spinal cord approached normal. Accordingly, Chd7 is necessary for the initiation of oligodendrocytic differentiation, and Chd7 loss causes a noticeable delay in the myelination of nerve fibers. A comparative analysis of gene expression profiles in the spinal cord between control mice and mice with the conditional Chd7 knockout (PND8) confirmed the importance of Chd7 for the regulation of genes responsible for the differentiation into OLs and for myelination; furthermore, in most cases (~84 %), it functions as a transcriptional activator (He et al., 2016).

Normally, in adult mice, Chd7 expression in the white matter of the spinal cord is practically undetectable; however, lysolecithin-induced demyelination causes local re-expression of Chd7 against the background of myelin regeneration via OPC recruitment. In the case of a Chd7 deletion in the affected tissue area, there was a significant reduction not only in the OL number but also in the expression levels of Mbp and Plp1 as compared to the control, and morphometric characteristics of myelinated fibers deteriorated. For these reasons, those authors believe that Chd7 is crucial for remyelination in case of whitematter damage (He et al., 2016). Chd7 has been reported to be a key regulator of OPC activation and proliferation after spinal cord injury (Doi et al., 2017).

In the expression regulation of the Chd7 gene itself, an important role is played by the Brg1 remodeler, the conditional knockout of which significantly suppresses Chd7 expression in mutant mice. Furthermore, within the Chd7 gene, multiple sites of cooperative binding of factors Brg1 and Olig2 have been identified, and these sites are functionally significant at the stage of OPCs and immature OLs (He et al., 2016)

A genome-wide search for Chd7-binding sites by ChIP-seq in differentiating OLs has shown that they are predominantly located in the region +5 kbp relative to a transcription start site of target genes and also serve as binding sites for transcription factor Olig2, which is known to regulate enhancers that are functionally important for oligodendrogenesis, in particular, by recruiting Brg1 to them (Yu et al., 2013; He et al., 2016).

A comparative analysis of distributions of ChIP-seq peaks for the remodelers Chd7 and Brg1 revealed that the peaks do not overlap in ~76 % of cases, and therefore Chd7 has unique molecular functions that control OL maturation (He et al., 2016).

CHD7 mutations cause human CHARGE syndrome, which is characterized by multiple pathologies including craniofacial anomalies, neurological dysfunction, and growth retardation. Most CHARGE patients show some degree of intellectual disability, and many have structural aberrations of the corpus callosum and cerebellar vermis (Martin, 2010).

Subfamily INO80/SWR

The remodeling complexes belonging to this subfamily can contain more than 10 subunits and perform a variety of functions, including facilitation of transcription activation and DNA repair (Clapier, Cairns, 2009). In one study (Elsesser et al., 2019), it was demonstrated that a member of this family, chromatin-remodeling complex TIP60/EP400, is an important component of the oligodendrocytic differentiation program. It turned out that a Cre-mediated knockout of Ep400 (core ATPase) at various oligodendrogenesis stages in transgenic mice does change the OPC number but is the reason for a sharp drop in the number of OLs that started terminal differentiation and initiated myelin gene expression.

Analysis of gene expression in the OLs from mice with the Ep400 knockout confirmed downregulation of not only terminal differentiation and myelination genes, such as Plp1, Mbp, Mog, and Nfasc, but also key components of the transcriptional network, e.g., Sox10, Nkx2.2, Olig2, Olig1, Myrf, and Fyn, the products of which regulate the processes of oligodendrocytic differentiation and myelination. In particular, in immature OLs, EP400 specifically binds to the promoter and enhancer ECR9 in the first intron of the Myrf gene and i) catalyzes the replacement of histone H2A by the specialized H2A.Z histone in many nucleosomes as well as ii) recruits the Sox10 transcription factor, with which EP400 physically interacts to activate Myrf expression during oligodendrocytic differentiation. After the differentiation, EP400 induces changes in the pattern of H2A.Z localization on the promoter.

Additionally, in cultured primary OLs, it was found that the absence of EP400 impairs repair processes, as evidenced by an increase in the amount of histone γH2A.X, which is a marker of a double-stranded DNA break and emerges as a result of phosphorylation of the specialized H2A.X histone. Because such breaks are a signal for apoptosis, it is possible to define EP400 as a factor that promotes OL survival and protects DNA from damage or helps with its repair (Elsesser et al., 2019). Given that upon completion of the differentiation into OLs and at initial stages of myelination, there is an increase in heterochromatinization and nuclear condensation (Mori, Leblond, 1970), it can be theorized that such critical changes in the structure of chromatin make it especially vulnerable during this period of oligodendrogenesis and require more attention from a “surveillance” system (Elsesser et al., 2019).

Thus, EP400 is not required for OPC specification or for early development of clones in embryogenesis but is necessary during terminal oligodendrocytic differentiation and the active phase of myelination.

## Conclusion

Each stage of oligodendrogenesis is accompanied by a change in the expression of a large number of genes. The activation of some genes and the repression of others are implemented by precisely spatially and temporally coordinated interactions of various transcription factors with the promoters and enhancers of these genes. Such fine regulation is possible due to the coordinated fine-tuned work of transcription factors and ATP-dependent chromatin-remodeling complexes, which determine the regulatory landscape of chromatin and contribute to its epigenetic modifications (Copray et al., 2009). In addition to many common properties among all chromatin remodelers, they have distinct features that explain their functional specialization in the cell (Hota, Bruneau, 2016), in particular, at different stages of differentiation in oligodendrogenesis. It has been shown that OP specification requires SWI/SNF complexes, which keep NSCs in an undifferentiated state until they respond to certain gliogenic signals. Besides, members of this subfamily are curators of transcriptional activity of genes at all stages of oligodendrocytic differentiation. By contrast, CHD7 is required for OPC proliferation, for the onset of oligodendrocytic differentiation, and for the activation of genes responsible for myelination and remyelination, whereas EP400 is necessary only for terminal oligodendrocytic differentiation and the active phase of myelination (Matsumoto et al., 2006, 2016; Yu et al., 2013; He et al., 2016; Doi et al., 2017; Elsesser et al., 2019).

The roles of ISWI subfamily members in oligodendrogenesis have not yet been investigated; however, given their functional feature – the assembly and positioning of nucleosomes (Hota, Bruneau, 2016; Clapier et al., 2017) – it is possible that they are important for heterochromatinization processes, which intensify with the gradual transition from OPCs to mature myelinating OLs, and for nuclear condensation (Mori, Leblond, 1970).

## Conflict of interest

The authors declare no conflict of interest.
